# The Hospital World

**Published:** 1910-04-23

**Authors:** 


### The Hospital World.

### Elections and Appointments.

Lord Castlereagh, M.P., has accepted the presidency of the Chelsea Hospital for Women.

Mr. H. B. Kenward and Mr. W. Bennett have been re-elected members of the committee of the Worcester Dispensary, on which the Rev. G. M. Isaac and Mr. Walter Wood are newly chosen.

Sir Gilbert Wills, Bart., has been re-elected president of the Royal Boscombe and West Hants Hospital. Viscount Portman, Mr. Walter Child Clark, Mrs. Maberly, and Mr. F. Moser have been re-elected vice-presidents.

Mr. Henry Clark, the Secretary of the Leicester Co-operative Society, has been nominated to a Life Governorship of the Leicester Infirmary in respect of the donation of £100 to the reconstruction scheme in commemoration of the Society's jubilee.

At the annual meeting of the governors of the Norfolk and Norwich Hospital the members of the board of management have been elected as follows :—County : Mr. Eustace Gurney, Mrs. S. Gurney Buxton, and Mr. Reginald Laurence. City : Archdeacon Pelham, Mr. F. C. Hinde, and Miss R. M. Gurney. The Bishop of Norwich has re-appointed the Rev. John Huxley chaplain to the Hospital, and Mr. Henry Birbeck has been again elected treasurer.

Mr. E. Gibson has been re-elected hon. secretary to the Royal Surrey County Hospital, and Mr. Powell hon. treasurer; Col. Lane, Mr. R. Bateman, Mr. C. J. Barlow, Mr. J. F. Chance, Mr. W. V. Cooper, Mr. J. Noon, Mr. H. J. Thomas, and Mr. H. Waddington have been re-elected to the committee. The new members to fill vacancies are Sir Henry Samuelson, the Rev. G. C. Fanshawe, Mr. W. T. Coleman, and Mr. J. W. G. Wollen.

### Personalia.

To Sit for Fifteen Years on a hospital committee speaks well for any man's enthusiasm, and a hard worker with other claims on his time may look justly at the end of that period for a respite. Recognition of this is to be found in the thanks given by the Surrey County Hospital Committee to Mr. Frank Lasham on his resigning his post on that body.

The Bedfordshire Hospital Guild held its annual meeting recently, when Lady Ampthill presided, supported by Lord Peel, Lord Ampthill, Miss Douglas Pennant, Mr. J. B. Hope, Mrs. Arthur Duberly (hon. sec.), Mr. H. C. Williams (hon. treasurer), Col. Hay Shaw, and Dr. Gifford Nash. The work of the guild was described by Lord Peel as follows : "The Guild began its work by instituting a machinery which divided the county into twenty divisions, and over nearly every one it placed a lady, and I think that is a wise course to pursue, because of those sympathies which qualify ladies for the position. The Guild helps the hospital by causing the county to take a common interest in a common object. The Guild has established that common interest." Lady Ampthill read the annual report, which stated that during the year the Guild had sent £114 to the hospital. Owing to various causes the following presidents of divisions have resigned : Capt. Higgins, Turvey; Mr. Braybrooks, Potton; Lady Osborne, Shefford; Mrs. Stobart, Oakley; Mrs. Talbot, Clophill; but Mrs. Archdale Sharpin, Mr. F. Gouldthorpe Smith, Dr. Temple, Mrs. F. Carpenter, and Mrs Argles have taken their places.

Several changes have recently been made in the managing body of the Kensington and Fulham General Hospital—formerly the Jubilee Hospital—an institution which has been regenerated. Lord Brassey, finding himself unable to give that personal service to the affairs of the institution which is demanded of the president of a voluntary hospital's board, has placed his resignation in the hands of the committee, and Lord Claud Hamilton, M.P., has been elected president. Lord Claud has taken a keen interest in hospital affairs, and has thrown himself vigorously into the work at the Fulham and Kensington, and he is likely to give as earnest personal service to this institution as his near relative, the Duke of Abercorn, gives to the West London. His Grace the Archbishop of Westminster, the Duke of Norfolk, Sir John French, the Earl of Kenmare, Sir Thomas Dewar, and Mr. Hayes Fisher have been elected vice-presidents. The Secretary, Mr. Louis McCausland, has worked very hard to revive an interest in the affairs of the institution, and we hope, in a future issue, to give some details of the work. Mr. McCausland is an old army man, who served in the Burmese campaign—medal and two clasps—and in 1899, on his retirement from active service, began to take an interest in institutional work, and joined the administrative staff of the Westminster Hospital as Steward. In 1905 he was selected as secretary of the Kensington and Fulham Hospital, at a period when the affairs of the institution were in a state of chaos. During the time he has been associated with the hospital he has been responsible for many improvements in the management and administration of the institution.

We are glad to record the appreciation which the Governors of the Royal Boscombe and West Hants Hospital have extended to Mr. A. Godwin Pratt on his resignation of the honorary secretaryship of the hospital. It was resolved at their annual meeting that : "This meeting of Governors and subscribers of the Royal Boscombe and West Hants Hospital, held on April 2, 1910, desires to record its appreciation of, and gratitude to, Mr. Godwin Pratt on his resignation of the office of hon. secretary to the hospital, an office he has filled with conspicuous ability and tact for many years past. It further directs that a copy of this minute, printed on vellum, and suitably framed, be handed to him as a small token of their esteem and respect." Sir Gilbert Wills, Bart., the president of the hospital, wrote : "Especially am I sorry to be absent on this occasion, as I am informed of the resignation of our indefatigable hon. secretary. I understand that he has decided to take this step owing to the ever-increasing volume of work which he now finds monopolises the whole of his time, but I am delighted to hear that he does not intend entirely to sever his connection with the hospital, and that we are still to have the benefit of his valuable advice and experience. . . . Though we all deplore the loss of Mr. Pratt, I think we may regard the increasing duties of the secretary as a marked indication of the expansion that the hospital has made, and is making." Mr. Pratt, who has been secretary for 17 years, in thanking the meeting, said : "I do not think my resignation should be viewed with any regret, for, as our President has remarked, it denotes the extent of the hospital's expansion, and my post is to be filled by a young and extremely able man." The new secretary, we understand, is Mr. Conrad Gilbert, son of the secretary of the West London Hospital, where he has gained a knowledge of a hospital secretary's work. Mr. Pratt is to be the first holder of the newly created post of vice-chairman, an honour he certainly deserves.

